# Ceftazidime-Avibactam plus aztreonam synergistic combination tested against carbapenem-resistant *Enterobacterales* characterized phenotypically and genotypically: a glimmer of hope

**DOI:** 10.1186/s12941-023-00573-3

**Published:** 2023-03-21

**Authors:** Rawan Taha, Ola Kader, Sherine Shawky, Shahinda Rezk

**Affiliations:** 1 Lecturer of Molecular and Diagnostic Microbiology, Microbiology Department, Medical Research Institute, Alexandria University, 165 Horreya Avenue, Hadara, Alexandria, Egypt; 2 Microbiology Department, Medical Research Institute, Alexandria, Egypt

**Keywords:** Carbapenem-resistant *Enterobacterales*, Carbapenemase types, Ceftazidime-Avibactam-Aztreonam, Synergy

## Abstract

**Background:**

Carbapenemase-producing *Enterobacterales* (CPE) show rapid global dissemination and pose a significant therapeutic challenge. This study aimed to characterize carbapenemase-producing *Klebsiella* spp. and *Escherichia coli* (*E. coli*) phenotypically and genotypically and evaluate the effect of ceftazidime/ avibactam plus aztreonam combination.

**Methods:**

A total of 219 *Klebsiella* species and 390 *E. coli* strains were isolated from clinical samples, in which 80 *Klebsiella* spp. and 20 *E coli* isolates were resistant to tested carbapenems (imipenem, ertapenem, meropenem) by disk diffusion/broth dilution method and Vitek-2 compact system. MASTDISCS *Combi Carba plus* discs and real time PCR were used to determine type of carbapenemase phenotypically and genotypically, respectively. Interestingly, the synergistic effect between ceftazidime-avibactam (E-test) and aztreonam (disc) was tested against the CPE isolates.

**Results:**

Out of the carbapenem-resistant isolates, 76.25% *Klebsiella* spp. isolates were extensively drug-resistant (XDR) while 18.75% were pan drug-resistant (PDR), and 5% were multidrug-resistant (MDR). Regarding *E. coli*, 5% were PDR, 20% were MDR and 75% were XDR. More than one carbapenemase gene was detected in 99% of the isolates. In comparison between MAST-*Carba plus* discs and PCR results, sensitivity and specificity were (85.42–97.92%) in *Klebsiella* spp., and (69.64–100%) in *E. coli*, respectively. Moreover, a strong association was detected between both test results among *Klebsiella* spp. (*p* < *0.001*) and *E. coli* (*p* = *0.012*) isolates. Finally, ceftazidime-avibactam and aztreonam combination showed a synergistic effect in 98.8% of *Klebsiella* spp. and 95% of *E coli.* All 16 PDR isolates showed synergy.

**Conclusion:**

This synergistic effect spots the light on new therapeutics for XDR and PDR CPE.

## Introduction

Antimicrobial resistance is recognized as one of the significant global health threats by the World Health Organization [1]. *E. coli* and *Klebsiella* spp. are considered to be the most important *Enterobacterales* that commonly cause health-associated and community-associated infections [2]. Among multidrug-resistant bacteria, carbapenemase-producing *Enterobacterales* (CPE) show rapid global dissemination and pose a significant therapeutic challenge [1, 3]. This is besides the high threat imposed by extensive drug resistant (XDR) and pan drug resistant (PDR) CPE. Multidrug-resistance is defined as resistance to at least one agent in three or more antimicrobial classes, XDR is defined as resistance to at least one agent in all but two or fewer antimicrobials, and PDR is defined as resistance to all agents in all antimicrobial classes [4].

Detection of carbapenemases rapidly is crucial to ensure the management and treatment of clinical infections [5]. These carbapenemases include Ambler class A (KPC*,* and GES enzymes) and class D (OXA-48-like enzymes) which are serine carbapenemases, and class B (Metallo carbapenemases); (NDM*,* VIM*,* and IMP enzymes) [6]. Phenotypic methods are commonly used in routine laboratory practice. These methods detect carbapenemase activity regardless of carbapenemase type, whereas inhibitor-based methods such as MASTDISCS *Combi Carba plus* disc (MAST-*Carba plus*; Mast Group Ltd., UK) can be used to differentiate between carbapenemase types [7]. However, PCR remains the gold standard for the detection of carbapenemase types [5].

With the advent of a new effective therapeutic option; ceftazidime-avibactam, a 3rd generation cephalosporin combined with a β-lactamase inhibitor, has a wide-spectrum activity against serine β-lactamases but is hydrolyzed by metallo β-lactamases [8]. In contrast, the aztreonam, monobactam, is stable in the presence of metallo β-lactamases but susceptible to hydrolysis by serine β-lactamases [3]. Thus, the combination of ceftazidime-avibactam and aztreonam is considered to have a synergistic therapeutic effect against CPE [9].

This study aims to evaluate the performance of MASTDISC *Combi Carba plus* discs for the detection of carbapenemase types and compare it to PCR results. Moreover, synergy between ceftazidime-avibactam plus aztreonam will be tested in highly resistant CPE.

## Materials and methods

### Bacterial isolates

A total of 219 *Klebsiella* species isolates and 390 *E. coli* isolates were identified by conventional biochemical methods then antibiotic susceptibility testing (AST) was performed using disc diffusion method at Microbiology Department, Medical Research Institute, Alexandria University between June 2021 and January 2022 [10]. Imipenem (10 µg), ertapenem (10 µg), and meropenem (10 µg) were used (Oxoid, Basingstoke, England).

Carbapenem-resistant isolates; 80 (36.53%) *Klebsiella* spp. and 20 (5.13%) *E. coli*; were subjected to full identification and AST to determine minimum inhibitory concentration (MIC) of carbapenems using VITEK^®^2 system (bioMérieux, France).

### Phenotypic detection of carbapenemase enzymes

MASTDISCS^®^ *Combi Carba plus* disc method (MAST-*Carba plus*; Mast Group Ltd., UK) was performed according to manufacturer’s instructions. The discs were placed on Muller-Hinton agar plate inoculated with 0.5 McFarland test strain. Plates were incubated at 37 °C for 18 to 24 h [7].

MAST-*Carba plus* consists of carbapenem alone (disc A) and in combination with a MBL inhibitor (disc B), a KPC inhibitor (disc C), an AmpC inhibitor (disc D), and temocillin in combination with a MBL inhibitor (disc E). The interpretation of the test is as follows: the zone of inhibition of the carbapenem disc (A) was compared to the zones of inhibition of each of the carbapenem plus inhibitor discs (B, C, and D). If disc B only shows a zone difference ≥ 5 mm than disc A, the organism was recorded as demonstrating MβL activity. If disc C only shows a zone difference ≥ 5 mm than disc A (D-A should be < 5 mm), the organism was recorded as demonstrating KPC activity. If discs C and D both show significant zone differences (≥ 5 mm) compared to disc A, the organism was recorded as demonstrating AmpC activity coupled with porin loss (impermeability). If disc E shows a zone of inhibition of ≤ 10 mm, the organism was recorded as demonstrating OXA-48 activity [7].

### Genotypic detection of carbapenemase genes

Bacterial DNA was extracted using Thermo-Scientific Gene-JET Genomic DNA Purification Kit (Thermo-Fisher, Vilnius, Lithuania) according to manufacturer’s instructions. Maxima SYBR Green qPCR Master Mix (2X) (Thermo-Fisher, Vilnius, Lithuania) was applied for detection of *bla*_NDM_*, bla*_VIM_*, bla*_IMP*,*_* bla*_KPC_*, bla*_GES_*,* and *bla*_OXA-48-like_ using Mx3000P™ Real Time PCR thermal cycler instrument (Stratagene, USA). A total of 6 µL of template DNA was added to the reaction mix which was composed of 10µL Maxima SYBR Green master mix and 0.6 µL for each forward and reverse primers mentioned in Table 1 [11–13] and 2.8 µL nuclease-free water. PCR started with an initial denaturation step at 95 °C for 10 min, followed by 45 cycles of DNA denaturation at 95 °C for 15 s, primer annealing at 55 °C for 30 s and primer extension at 72 °C for 30 s. Afterward, dissociation curve analysis was performed, consisting of 1 cycle at 95 °C for 1 min, then 55 °C for 30 s, and finally 95 °C for 30 s.

**Table 1:** Primer sequences and amplicon sizes

Primer	Sequence	Gene	Expected product size, bp	References
KPC-F	CGTCTAGTTCTGCTGTCTTG	*bla*_KPC_	232	[11]
KPC-R	CTTGTCATCCTTGTTAGGCG			
GES-F	CTATTACTGGCAGGGATCG	*bla*_GES_	594	[13]
GES-R	CCTCTCAATGGTGTGGGT			
NDM-F	GGTTTGGCGATCTGGTTTTC	*bla*_NDM_	621	[12]
NDM-R	CGGAATGGCTCATCACGATC			
IMP-F	GGAATAGAGTGGCTTAAYTC	*bla*_IMP_	232	[11]
IMP-R	TCGGTTTAAYAAAACAACCACC			
VIM-F	GATGGTGTTTGGTCGCATA	*bla*_VIM_	390	[11]
VIM-R	CGAATGCGCAGCACCAG			
OXA-48-F	GCGTGGTTAAGGATGAACAC	*bla*_OXA-48_	438	[11]
OXA-48-R	CATCAAGTTCAACCCAACCG			

### E-test-Disc diffusion modified method

Ceftazidime-avibactam (CAZ-AVI) E-test (Liofilchem^®^, Italy), containing CAZ (0.016–256 µg/mL)—AVI (4 µg/mL), and aztreonam (ATM) disc (30 µg, Oxoid, Basingstoke, England) were used to determine CAZ-AVI and ATM synergy for the 100 isolates in triplicates. After adjustment to 0.5 McFarland, the tested bacterial suspension was uniformly streaked over the surface of Muller Hinton agar plates. A CAZ-AVI E-test strip was placed 15 mm from the disc such that the center of the disc was placed parallel to the sensitivity breakpoint of the CAZ-AVI E-test (8 µg/mL). Plates were incubated at 37 °C for 16 to 18 h. Two approaches were used to identify synergy, the method used depended on the pattern detected. First, the presence of an inverse-D was interpreted as synergy, and that was a qualitative approach. Second, a quantitative approach was used as the zone radius for ATM disc alone was measured, and MIC for CAZ/AVI was read using breakpoints from CLSI M100 [3, 10].

### Statistical analysis of the data

Data were fed to the computer and analyzed using IBM SPSS software package version 20.0*.* (Armonk, NY: IBM Corp) Qualitative data were described using numbers and percentages. Significance of the obtained results was judged at the 5% level (*p* < 0.05). The used tests were chi-square test, for categorical variables, to compare different groups, and Fisher’s Exact in correction for chi-square when more than 20% of the cells have an expected count of less than 5.

The sensitivity was calculated from the number of MBLs-, KPC-, and OXA-48-possessing organisms that were correctly determined. The specificity was calculated from the number of non-MBLs-, non-KPC- or non-OXA-48-possessing organisms that were correctly determined. The positive predictive value (PPV) was calculated from the probability that MBLs-, KPC-, and OXA-48-possessing organisms detected by MAST-*Carba plus* indeed do possess these enzymes. The negative predictive value (NPV) was calculated from the probability that non-MBLs-, non-KPC- or non-OXA-48-possessing organisms detected by MAST-*Carba plus* indeed do not possess these enzymes.

## Results

A total of 219 *Klebsiella* species and 390 *E. coli* strains were isolated from clinical samples, in which 80 *Klebsiella* spp. *and* 20* E. coli* were found to be carbapenem-resistant. Among the 80 carbapenem-resistant *Klebsiella* species isolates, 79 (98.75%) were identified as *Klebsiella pneumoniae* ssp *pneumoniae* while 1 *Klebsiella* species (1.25%) was identified as *Klebsiella pneumoniae* ssp *ozaenae.* The majority of *Klebsiella* species were isolated from blood cultures 47 (58.8%) while the majority of *E. coli* were isolated from aspirate 7 (35%). Distribution of the isolates among clinical specimens is shown in Table 2.

**Table 2:** Distribution of carbapenem-resistant *Klebsiella* species and *E. coli* among clinical specimens

Specimen	*Klebsiella* species isolates		*E. coli* isolates		Total number of isolates
	№	%	№	%	
Blood cultures	47	58.8	4	20	51
Wound swabs	13	16.3	2	10	15
Urine	7	8.8	6	30	13
Fluid aspirate	4	5	7	35	11
Sputum	6	7.5	0	0	6
Bronchoalveolar lavage (BAL)	2	2.5	0	0	2
Central venous catheter (CVC)	1	1.3	0	0	1
Pus aspirate	0	0	1	5	1
Total	80	100	20	100	100

### Antimicrobial susceptibility testing

**Fig. 1:**
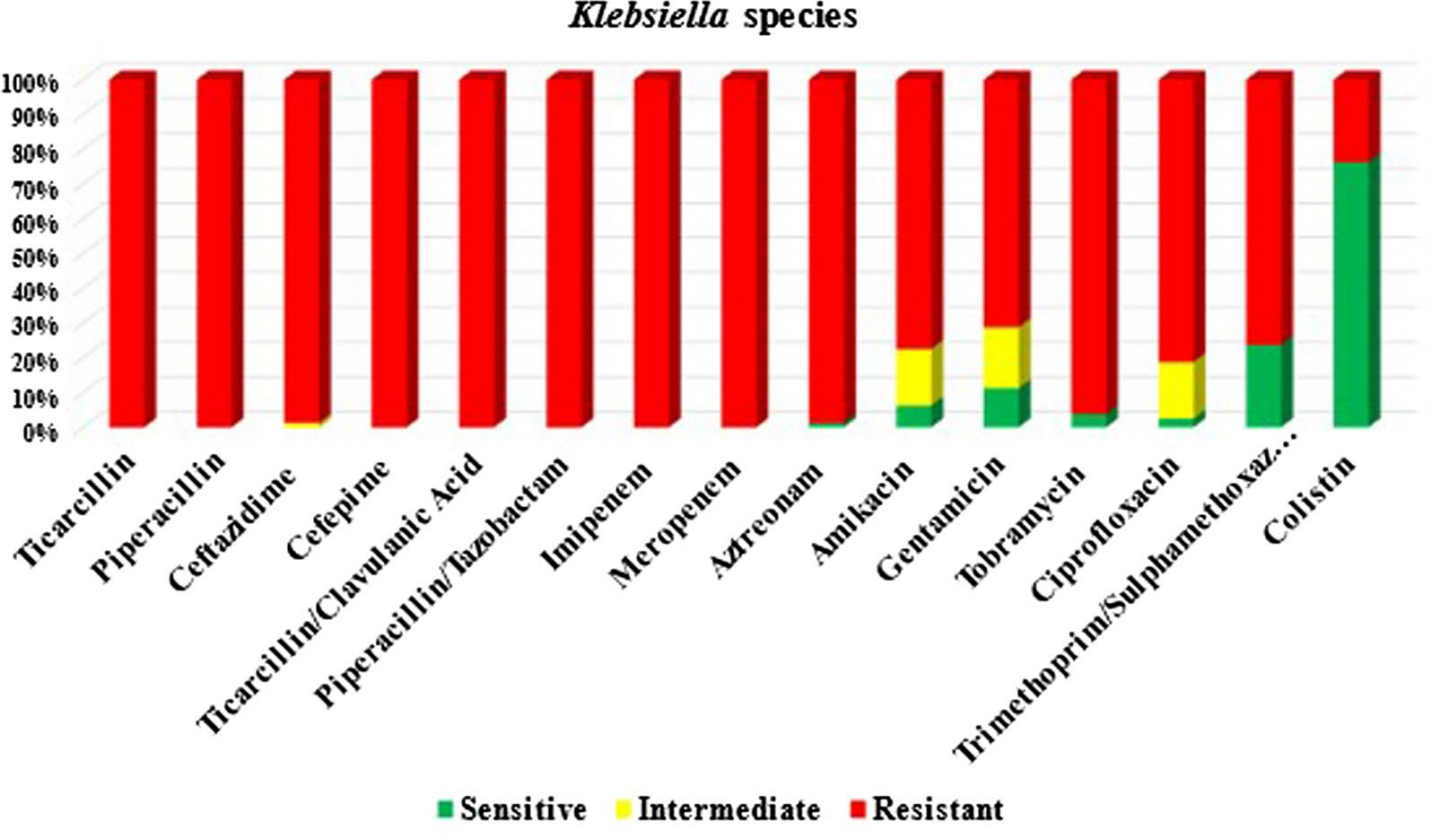
Antimicrobial susceptibility testing of carbapenem-resistant *Klebsiella* species isolates

All 80 carbapenem-resistant *Klebsiella* species isolates were resistant to penicillins and cephalosporins group, 79 (98.75%) isolates were resistant to aztreonam. High resistance rates were detected for aminoglycosides, quinolones, and sulfonamides. In that, 77 (96.25%) isolates were resistant to tobramycin, 62 (77.5%) isolates were resistant to amikacin, 57 (71.25%) isolates were resistant to gentamycin, 65 (81.25%) isolates were resistant to ciprofloxacin, and 61 (76.25%) isolates were resistant to sulfonamides. Colistin had the lowest resistance rate 19 (23.75%), as shown in Fig. 1.

**Fig. 2:**
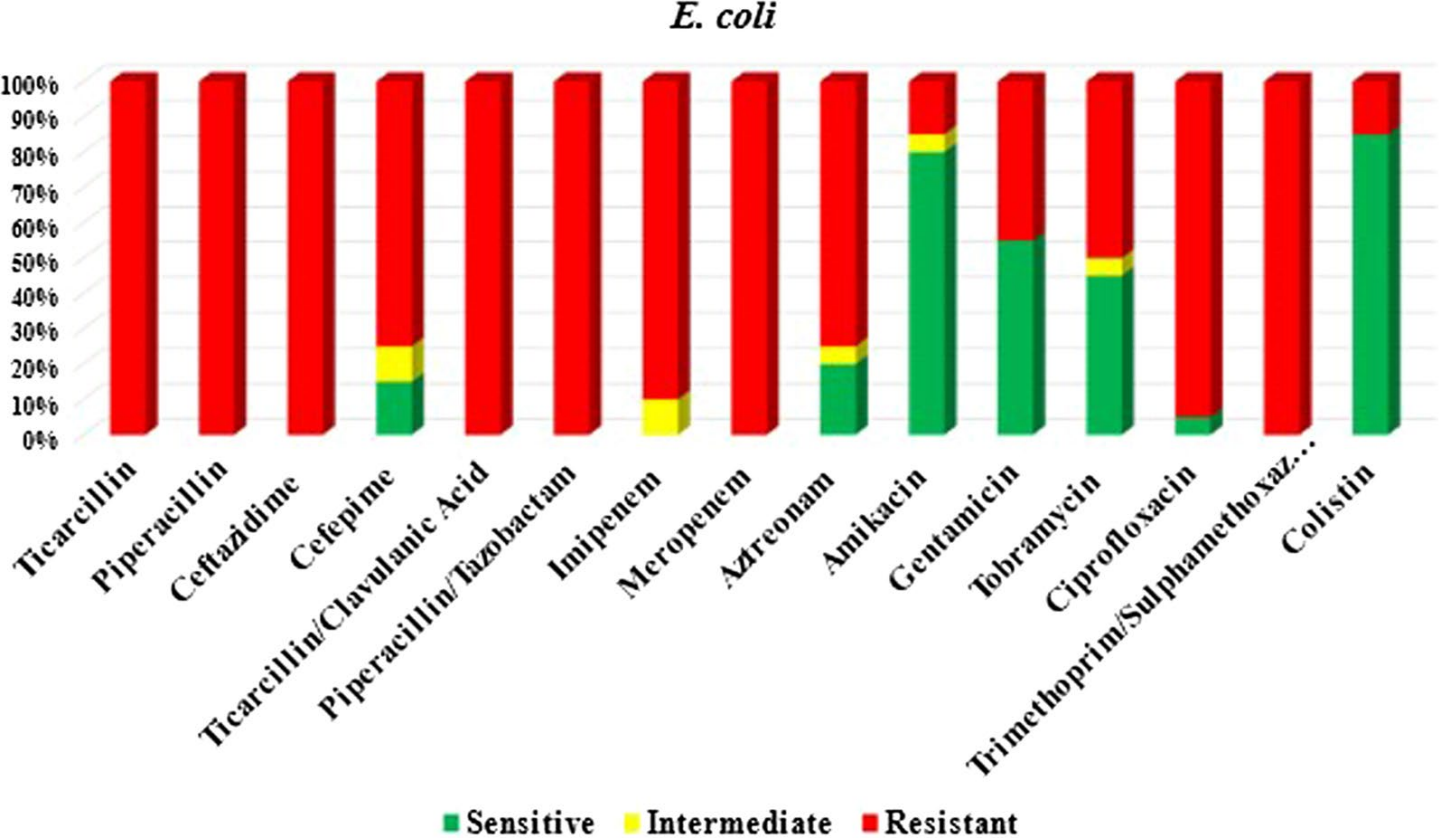
Antimicrobial susceptibility testing of carbapenem-resistant *E. coli* isolates

All the 20 carbapenem-resistant *E. coli* isolates were resistant to the penicillin group, ceftazidime, β-lactam/β-lactamase inhibitors, and sulfonamides. Nineteen (95%) isolates were resistant to ciprofloxacin, 15 (75%) isolates were resistant to cefepime and aztreonam, and only 3 (15%) were resistant to colistin. Ten (50%) isolates were resistant to tobramycin, 9 (45%) isolates were resistant to gentamycin, and 3 (15%) isolates were resistant to amikacin, as shown in Fig. 2.

The majority of the *Klebsiella* spp. isolates were XDR (76.25%), while 18.75% were PDR, and only 5% were MDR. On the other hand, only 5% *E. coli* showed pan drug-resistance, while the majority were either MDR (20%) or XDR (75%).

### Prevalence of carbapenemase genes

Carbapenemase production was detected in all (100%) *Klebsiella* species and *E. coli,* presented in Table 3. Among the 80 *Klebsiella* isolates, the most common gene identified was *bla*_NDM_ (91.25%). On the other hand, *bla*_VIM_ was detected in all *E. coli* isolates 100%. *bla*_IMP_ wasn’t detected in both genera.

**Table 3:** Genotypic detection of carbapenemase genes among carbapenem-resistant *Klebsiella* species and *E. coli* isolates

Carbapenemase genes	*Klebsiella* species (n = 80)		*E. coli* (n = 20)		χ^2^	P
	No	%	No	%		
*bla*_KPC_	9	11.25%	17	85%	45.231^*^	< 0.001^*^
*bla*_GES_	56	70%	18	90%	3.326	0.068
*bla*_NDM_	73	91.25%	19	95%	0.306	^FE^p = 1.000
*bla*_IMP_	0	0.0%	0	0.0%	–	–
*bla*_VIM_	55	68.75%	20	100%	8.333^*^	0.004^*^
*bla*_OXA-48_	58	72.5%	19	95%	4.574^*^	0.038^*^

χ^2^: Chi square test FE: Fisher Exact

p: p value for comparing between *Klebsiella* species and *E. coli*

^*^: Statistically significant at p ≤ 0.05

### Phenotypic detection of carbapenemase enzymes by MAST-*Carba plus*

**Fig. 3:**
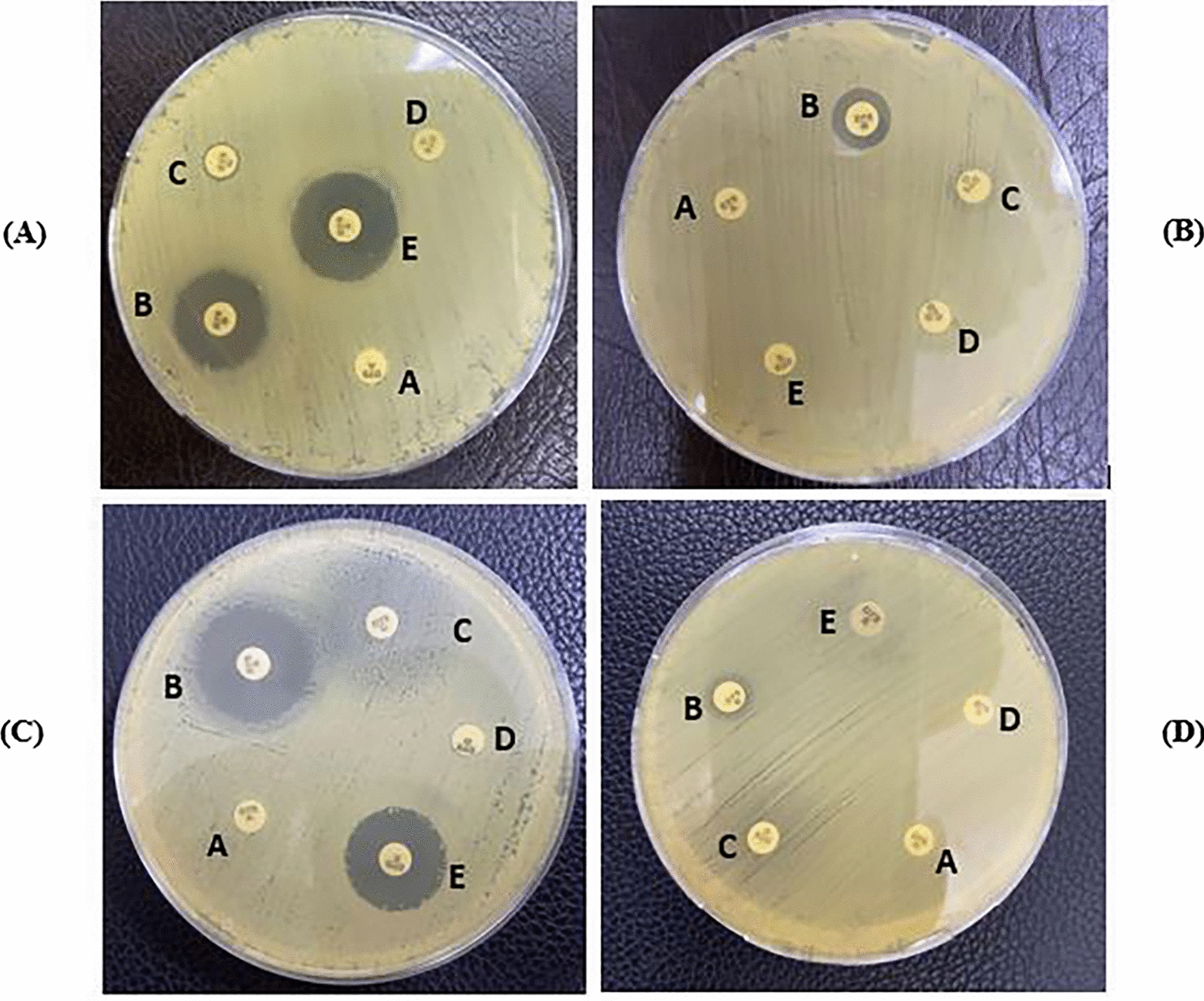
Results of MAST-*Carba plus* in detection of carbapenemase-producing isolates. **A** Shows a zone of inhibition of ≥ 5 mm around disc B compared to that of disc A indicating MβL activity, while discs C and D show no zones of inhibition differences compared to that of disc A indicating absence of KPC and AmpC activities and disc E shows a zone of inhibition of > 10 mm indicating absence of OXA-48 activity. **B** Shows a zone of inhibition of ≥ 5 mm around disc B compared to that of disc A indicating MβL activity, and disc E shows a zone of inhibition ≤ 10 mm indicating OXA-48 activity, while discs C and D show no zones of inhibition differences compared to that of disc A indicating absence of KPC and AmpC activities. **C** Shows a zone of inhibition of ≥ 5 mm around disc B compared to that of disc A indicating MβL activity, disc C shows a reduction of growth around the disc which considered to be indicative for KPC activity while disc D shows no zone of inhibition differences compared to that of disc A indicating absence of AmpC activity, and disc E shows a zone of inhibition > 10 mm indicating absence of OXA-48 activity. **D** Shows a zone of inhibition of ≤ 10 mm around disc E indicating OXA-48 activity, while discs B, C and D show no differences in zones of inhibition compared to that of disc A indicating absence of MβL, KPC and AmpC activities

PCR results were the gold standard to evaluate the performance of MAST-*Carba plus* for detection of carbapenemase enzymes. Table 4 and Fig. 3 show the results of MAST-*Carba plus* for detection of carbapenemase enzymes.

**Table 4:** Pattern of distribution of carbapenemase enzymes by using MAST-*Carba plus*

*Klebsiella* species (n = 80)		*E. coli* (n = 20)	
Carbapenemase enzymes	Total no. of isolates (%)	Carbapenemase enzymes	Total no. of isolates (%)
OXA-48*,* and Metallo β-lactamases	38 (47.5%)	Metallo β-lactamases, and KPC	6 (30%)
Metallo β-lactamases	30 (37.5%)	Metallo β-lactamases	5 (25%)
OXA-48*,* Metallo β-lactamases, and KPC	4 (5%)	OXA-48*,* and Metallo β-lactamases	4 (20%)
OXA-48	4 (5%)	OXA-48*,* Metallo β-lactamases, and KPC	3 (15%)
None	2 (2.5%)	OXA-48*,* and KPC	1 (5%)
Metallo β-lactamases, and KPC	1 (1.25%)	OXA-48*,* Metallo β-lactamases, KPC*,* and Amp C	1 (5%)
KPC	1 (1.25%)

For *Klebsiella* species isolates, the sensitivity of the MAST-*Carba plus* for KPC producers was 55.56%, the specificity was 98.60%, the PPV was 83.33% and the NPV was 94.60%. For MBL producers, the sensitivity was 93.51%, the specificity was 66.67%, the PPV was 98.63% and the NPV was 28.57%. For OXA-48 producers, the sensitivity was 79.31%, the specificity was 100%, the PPV was 100% and the NPV was 64.71%.

For *E. coli* isolates, the sensitivity of the MAST-*Carba plus* for KPC producers was 64.71%, the specificity was 100%, the PPV was 100% and the NPV was 33.33%. For MBL producers, the sensitivity was 95%, the specificity of test was 0.00%, the PPV was 100% and the NPV was 0.00%. For OXA-48 producers, the sensitivity was 47.37%, the specificity of test was 100%, the PPV was 100% and the NPV was 9.10%. The true positive, true negative, false positive, and false negative values of MAST-*Carba plus* in comparison to PCR results were shown in Table 5.

**Table 5:** True positive, true negative, false positive, and false negative values of MAST-*Carba plus* in comparison to PCR results

*Klebsiella* species isolates					*E. coli* isolates				
KPC		Genotypic		Total	KPC		Genotypic		Total
		Positive	Negative				Positive	Negative	
		No	No				No	No	
Phenotypic	Positive	5	1	6	Phenotypic	Positive	11	0	11
	Negative	4	70	74		Negative	6	3	9
Total		9	71	80	Total		17	3	20
MBL		Genotypic		Total	MBL		Genotypic		Total
		Positive	Negative				Positive	Negative	
		No	No				No	No	
Phenotypic	Positive	72	1	73	Phenotypic	Positive	19	0	19
	Negative	5	2	7		Negative	1	0	1
Total		77	3	80	Total		20	0	20
OXA-48		Genotypic		Total	OXA-48		Genotypic		Total
		Positive	Negative				Positive	Negative	
		No	No				No	No	
Phenotypic	Positive	46	0	46	Phenotypic	Positive	9	0	9
	Negative	12	22	34		Negative	10	1	11
Total		58	22	80	Total		19	1	20

The highest sensitivity in the present study was for MBLs detection in *E. coli* (95%) and the lowest was for KPC detection in *Klebsiella* isolates (55.56%). There was a strong statistically significant association detected between PCR and MAST-*Carba plus* results among *Klebsiella* spp. (*p* < *0.001*) and *E. coli* (*p* = *0.012*) isolates.

### Ceftazidime-Avibactam and aztreonam synergy testing

For *Klebsiella* spp. isolates, synergy was observed between CAZ/AVI E- test and ATM disc in 79 (98.75%) out of 80 isolates, 76 (95%) isolates were resistant to both CAZ/AVI alone and ATM alone, while 3 (3.75%) isolates were susceptible to CAZ/AVI E-test (MIC = 1.5 µg/mL) but resistant to ATM disc. No synergy was detected in 1 (1.25%) isolate, which was susceptible to ATM disc alone (26 mm).

For *E. coli* isolates, synergy was observed between CAZ/AVI E-test and ATM disc in 19 (95%) out of the 20 isolates, 14 (70%) isolates were resistant to both CAZ/AVI alone and ATM alone, while 4 (20%) isolates were susceptible to ATM disc but resistant to CAZ/AVI E-test and 1 (5%) isolate was susceptible to CAZ/AVI E-test (MIC = 1.5 µg/mL) but resistant to ATM disc. One (5%) isolate was resistant to both ATM disc and CAZ/AVI E-test with no synergy observed between them.

**Fig. 4:**
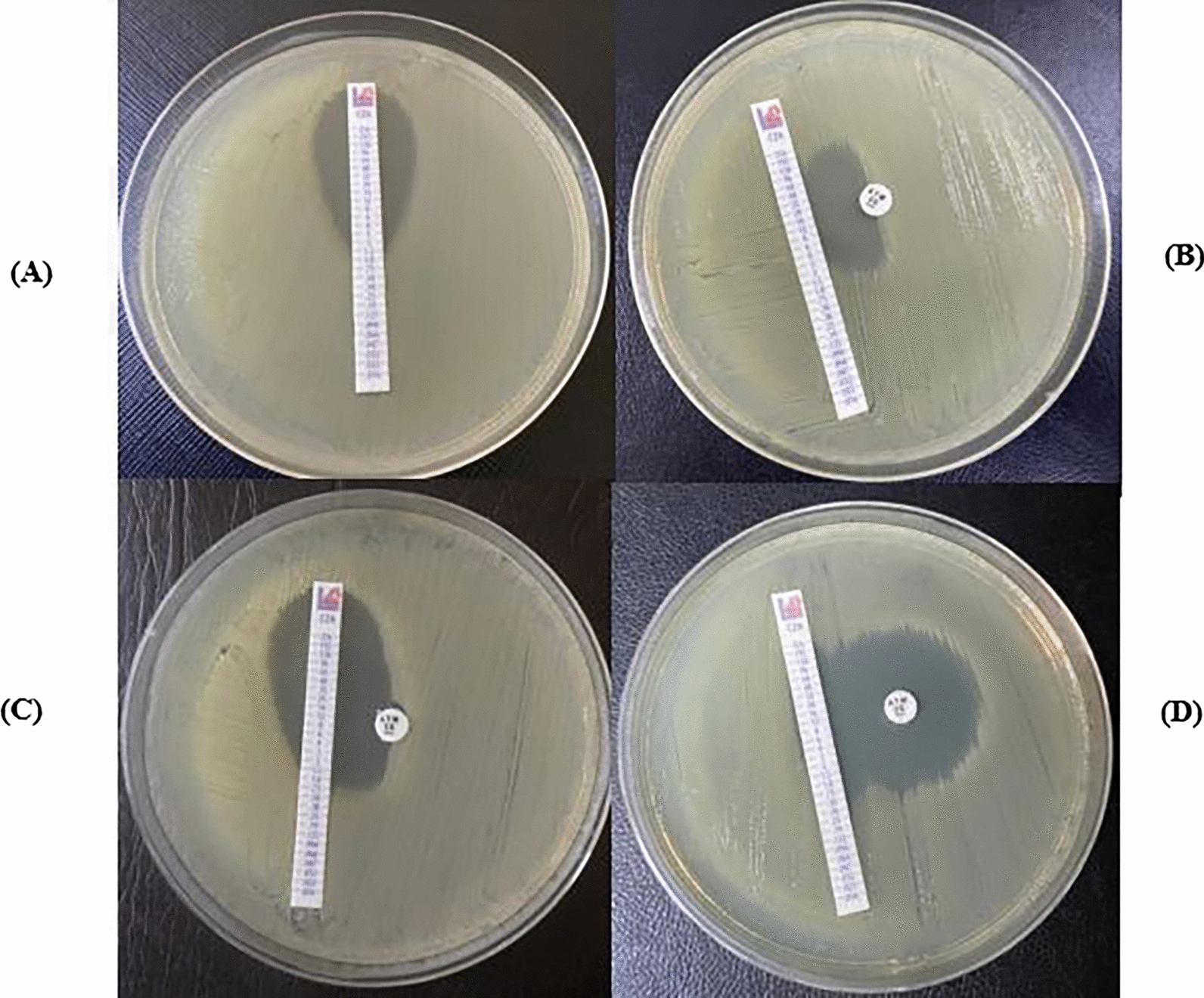
Pattern of susceptibility between CAZ-AVI E-test and ATM disc. **A** Shows MIC reading to CAZ/AVI alone (MIC = 1.5 µg/mL, reported as 2 µg/mL) **B** Shows inverse D-zone demonstrating the synergy between CAZ/AVI E-test and ATM disc (**C**) Shows susceptibility to CAZ/AVI E-test alone and synergy between CAZ/AVI E-test and ATM disc (**D**) Shows susceptibility to ATM disc with 30 mm zone diameter and synergy between CAZ/AVI E-test and ATM disc

All the pan drug-resistant tested isolates showed synergy between CAZ/AVI E-test and ATM disc. Different synergy patterns are shown in Fig. 4.

## Discussion

Carbapenems are often considered the last-line antibiotics used for the treatment of MDR *Enterobacterales* infections since they are stable even in response to ESBLs and AmpC enzymes. However, carbapenem resistance is progressively reported among *Enterobacterales* in recent years. Therefore, the rapid spread of CPE is considered to be a serious global health problem, and the early detection of CPE is crucial for targeted antimicrobial therapy and for timely and effective infection control [5].

This study detected a highly statistically significant difference between the prevalence of carbapenem-resistance among *Klebsiella* spp. isolates (80/219, 36.53%) and *E. coli* isolates (20/390, 5.13%), (*p* < 0.001). Several recent studies have reported similar results, in which carbapenem resistance was more prevalent in *Klebsiella* spp. than *E. coli* [8, 14–19].

The prevalence of β-lactam resistant *Klebsiella* spp. and *E. coli* isolates is increasing gradually. This may be due to misuse of antibiotics and repeated exposure to β-lactam drugs such as penicillin, third and fourth-generation cephalosporins, and carbapenems [20].

Extensively and pan drug-resistant isolates are increasingly being detected globally. The development of pan drug-resistant isolates is worrisome because they are practically not affected by any antibiotic, therefore higher rates of morbidity and mortality are expected. Regarding carbapenem-resistant *Klebsiella* spp. isolated in this study, 76.25% were XDR, 18.75% were PDR, and only 5.00% were MDR, while among *E. coli* isolates, 75% were XDR, 20% were MDR, and 5% showed PDR. An alarming elevated prevalence of MDR, XDR, and PDR isolates was also revealed by many up-to-date studies [20–23].

The carbapenemase genes are mainly located on mobile genetic elements, facilitating their spread among different bacteria [13]. The prevalence of carbapenemase genes is highly affected by geographic region and has been associated with serious epidemics that represent a major therapeutic challenge [24].

In light of this, rapid detection of CPE producers might be the best way to prevent their spread. PCR is always used as the gold standard technique for the detection of carbapenemase genes [11]. In the current study and in another Egyptian study [25], *bla*_NDM_ gene followed by *bla*_OXA-48_ gene showed the highest prevalence in the *Klebsiella* isolates, while *bla*_IMP_ gene was not detected among all isolates.

Furthermore, other studies in different geographical regions reported different gene distributions in *Klebsiella* spp. isolates. In a European survey, Grundmann et al. [14] reported that the most frequently detected carbapenemases were KPC enzymes. Consistent results were also declared by Han et al. [26] in China. Furthermore, in Iraq; Haji et al. [20] reported *bla*_VIM_ gene to be the most prevalent gene detected and no *bla*_KPC_ gene was found. Regarding Saudi Arabia, Alhazmi et al. [27]found that *bla*_OXA-48_ gene was the most common gene detected, and consistent with our results all of their isolates were negative for the *bla*_IMP_ gene.

In the present study, *bla*_VIM_ gene was detected all carbapenem-resistant *E. coli* isolates, whereas *bla*_IMP_ gene was not detected at all. Variations in the distribution of the carbapenemase genes was also recognized among the *E. coli* isolates in various countries [14, 20, 26, 28–33].

More than one carbapenemase gene was detected among 79/80 *Klebsiella* isolates and in all *E. coli* isolates. These findings were similarly stated by other researchers [18, 20, 30, 31, 34–36]. Hereby, the high prevalence of more than one carbapenemase gene in *Enterobacterales* isolates may illustrate the hastily propagating resistance. Consequently, determination of the mobile carbapenemase genes became an urge to contain this elevating resistance and to apply strict infection control measures in order to save lives.

In developing countries, many laboratories have no accessible PCR instruments due to its high cost. Accordingly, this study aimed to evaluate MAST*-Carba plus* in relation to PCR as a gold standard for the detection of carbapenemase-producing *Klebsiella* species and *E. coli* isolates. A statistically significant association was found between MAST-*Carba plus* and PCR results regarding detection of carbapenemase enzymes among carbapenem-resistant *Klebsiella* species (*p* < *0.001*), and among carbapenem-resistant *E. coli* isolates (*p* = *0.012*). We agree with Doyle et al. [37] who stated that if a clinical laboratory can’t afford to perform molecular tests, MAST*-Carba plus* can be used to confirm the presence of carbapenemase.

Resistance to safe and broad-spectrum drugs such as carbapenems often poses a challenge in antibiotic therapy, especially for MBLs producing-bacteria. Due to the very high level of resistance and high prevalence of carbapenemase genes, one of our main objectives was to evaluate a new synergetic combination between CAZ/AVI and ATM that would treat carbapenem-resistant *Enterobacterales.* Promising results were revealed. Synergy was observed between CAZ/AVI E- test and ATM disc in 79 (98.75%) out of the 80 carbapenem-resistant *Klebsiella* species isolates, and in 19 (95%) out of the 20 carbapenem-resistant *E. coli* isolates. A glimmer of hope emerged when all the 16 PDR showed a synergistic effect.

Few recent studies had also demonstrated the synergy between CAZ/AVI and ATM in MDR, XDR and PDR isolates by using different testing techniques. [3, 38–42].

In the present study, among the 100 carbapenem-resistant *Klebsiella* species and *E. coli* isolates, 90 (90%) were resistant to both CAZ/AVI E-test alone and ATM disc alone but showed synergy between CAZ/AVI and ATM, these isolates produce both metallo β-lactamases and serine β-lactamases. Similarly, Jayol et al. [43] who used E-test strips method reported that the combination of CAZ/AVI and ATM was synergistic for all their 63 isolates, and stated that this combination is effective against MBL-producing *Klebsiella pneumoniae* and particularly against isolates producing more than one carbapenemase genes. Therefore, the combination of CAZ/AVI and ATM is considered an effective therapeutic option particularly against *Klebsiella* species and *E. coli* isolates producing more than one carbapenemase gene of metallo β-lactamases and serine β-lactamases.

However, in vivo efficacy and safety of this regimen have to be evaluated. To the best of our knowledge, there was only one recent study conducted by Falcone et al. [9] to clinically compare the effect of CAZ/AVI and ATM combination to other active antibiotics on the outcome of patients with bloodstream infections due to MBL-producing *Enterobacterales* and showed that the treatment with CAZ/AVI and ATM was linked with lesser clinical failure at day 14, lower mortality at day 30, and shorter length of hospitalization.

## Conclusion

MAST-*Carba plus* is recommended to be used as a phenotypic screening method for detecting the carbapenemase enzymes in laboratories with no molecular resources. The enhanced activity observed with the combination of CAZ/AVI and ATM, compared to either agent alone, is promising and requires us to carefully consider the combination of CAZ/AVI and ATM as a new therapeutic option to treat infections caused by highly resistant *Enterobacterals* strains. Nevertheless, we are in need for more in vivo studies.

## Data Availability

The datasets used and/or analysed during the current study are available from the corresponding author on reasonable request.
