# Correction: Ceftazidime-Avibactam plus aztreonam synergistic combination tested against carbapenem-resistant *Enterobacterales* characterized phenotypically and genotypically: a glimmer of hope

**DOI:** 10.1186/s12941-023-00578-y

**Published:** 2023-04-18

**Authors:** Rawan Taha, Ola Kader, Sherine Shawky, Shahinda Rezk

**Affiliations:** 1 Microbiology Department, Medical Research Institute, Alexandria University, Alexandria, Egypt; 2 Lecturer of Molecular and Diagnostic Microbiology, Microbiology Department, Medical Research Institute, Alexandria University, 165 Horreya Avenue Hadara, Alexandria, Egypt

**Correction: Ann Clin Microbiol Antimicrob (2023) 22:21**

10.1186/s12941-023-00573-3

Following publication of the original article [1], the author noticed the errors in the affiliation details. The first author, “Rawan Taha” should be affiliated only in “Microbiology Department, Medical Research Institute, Alexandria University, Egypt”. The right affliations for the authors should be as follows.

**Rawan Taha (1)**

**Ola Kader (1)**

**Sherine Shawky (1)**

**Shahinda Rezk (2)**

**1**: Microbiology Department, Medical Research Institute, Alexandria University, Egypt.

**2**: Lecturer of Molecular and Diagnostic Microbiology, Microbiology Department, Medical Research Institute, Alexandria University, 165 Horreya Avenue, Hadara, Alexandria, Egypt.

The original article has been corrected.
